# Cross-sectional networks of depressive symptoms before and after antidepressant medication treatment

**DOI:** 10.1007/s00127-018-1506-1

**Published:** 2018-04-07

**Authors:** Fionneke M. Bos, Eiko I. Fried, Steven D. Hollon, Laura F. Bringmann, Sona Dimidjian, Robert J. DeRubeis, Claudi L. H. Bockting

**Affiliations:** 1 Department of Psychiatry, Rob Giel Research Center, University Medical Center Groningen, University of Groningen, PO Box 30.001, 9700 RB Groningen, The Netherlands; 2 Department of Psychology, University of Pennsylvania, Philadelphia, PA USA; 3 Department Quantitative Psychology and Individual Differences, University of Leuven, Leuven, Belgium; 4 Department of Psychology, University of Amsterdam, Amsterdam, The Netherlands; 5 Department of Psychology, Vanderbilt University, Nashville, TN USA; 6 Department of Psychology and Neuroscience, University of Colorado Boulder, Boulder, CO USA; 7 Department of Psychiatry, Amsterdam Medical Center, University of Amsterdam, Amsterdam, The Netherlands

**Keywords:** Selective serotonin reuptake inhibitors, Major depressive disorder, Network analysis, Gaussian graphical models (GGM), Antidepressant treatment

## Abstract

**Purpose:**

Recent reviews have questioned the efficacy of selective serotonin reuptake inhibitors (SSRIs) above placebo response, and their working mechanisms remain unclear. New approaches to understanding the effects of SSRIs are necessary to enhance their efficacy. The aim of this study was to explore the possibilities of using cross-sectional network analysis to increase our understanding of symptom connectivity before and after SSRI treatment.

**Methods:**

In two randomized controlled trials (total *N* = 178), we estimated Gaussian graphical models among 20 symptoms of the Beck Depression Inventory-II before and after 8 weeks of treatment with the SSRI paroxetine. Networks were compared on connectivity, community structure, predictability (proportion explained variance), and strength centrality (i.e., connectedness to other symptoms in the network).

**Results:**

Symptom severity for all individual BDI-II symptoms significantly decreased over 8 weeks of SSRI treatment, whereas interconnectivity and predictability of the symptoms significantly increased. At baseline, three communities were detected; five communities were detected at week 8.

**Conclusions:**

Findings suggest the effects of SSRIs can be studied using the network approach. The increased connectivity, predictability, and communities at week 8 may be explained by the decrease in depressive symptoms rather than specific effects of SSRIs. Future studies with larger samples and placebo controls are needed to offer insight into the effects of SSRIs.

**Trial Registration:**

The trials described in this manuscript were funded by the NIMH.

*Pennsylvania/Vanderbilt study*:

5 R10 MH55877 (https://projectreporter.nih.gov/project_info_description.cfm?aid=6186633&icde=28344168&ddparam=&ddvalue=&ddsub=&cr=1&csb=default&cs=ASC&MMOpt=).

*Washington study*:

R01 MH55502 (https://projectreporter.nih.gov/project_info_description.cfm?aid=2034618&icde=28344217&ddparam=&ddvalue=&ddsub=&cr=5&csb=default&cs=ASC).

**Electronic supplementary material:**

The online version of this article (10.1007/s00127-018-1506-1) contains supplementary material, which is available to authorized users.

## Introduction

Currently, selective serotonin reuptake inhibitors (SSRIs) are recommended as the first treatment of choice by the American Psychiatric Association (APA) and the National Institute of Clinical Excellence (NICE) for patients with major depressive disorder (MDD) [1, 2]. However, recent reviews have questioned the efficacy of SSRIs [3–5]. Given that there is a substantial proportion of patients who do not seem to benefit from acute antidepressant treatment [6], it is important to increase our understanding of the way SSRIs work so as to enhance their efficacy.

A relatively novel conceptual and statistical approach may provide an interesting framework to study the working mechanisms of SSRIs. The network approach conceptualizes symptoms of depression as part of a network of co-occuring symptoms that interact to create the symptom profile that is termed depression [7]. Instead of assuming that symptoms passively originate from a common cause (e.g., depression), the network approach proposes a focus on how individual symptoms are related to each other [8]. The network approach is thus fundamentally different from the routine modus operandi of monitoring treatment response at the aggregate level based on a large number of disparate psychiatric symptoms that are added up to a sum-score, which may obfuscate important insights [9]. The network approach also can help to identify ‘central’ symptoms—symptoms that often co-occur with other symptoms and thus are assumed to easily trigger or be triggered by other symptoms [10].

Prior studies that have used network analysis to characterize symptom patterns in depressed patients have yielded interesting results. Loss of energy and anhedonia appear to be central symptoms in analyses of both cross-sectional data and dynamic data using experience sampling methodology (ESM), the latter of which allows for the study of predictive effects of the symptoms on each other [11–13]. Further, depressed patients appear to have increased network connectivity or denser networks in cross-sectional data when compared to remitted patients [14] and in dynamic data when compared to healthy controls [15], suggesting that once one symptom is present, other symptoms might be easily triggered. However, in studies following patients at consecutive time points, the opposite pattern has been found: increased network connectivity when depressive symptom severity decreases [16].

With regard to potential effects of antidepressant treatment, only two studies examined the effects of antidepressants on the network structure of depressive symptoms. A recent study using dynamic data could not find evidence that the tricyclic antidepressant imipramine changed the dynamic associations between mental states [17]. In the STAR*D sample, using cross-sectional data, sad mood was found to be the most central symptom after several weeks of treatment with the SSRI citalopram [18]. However, the absence of a pretreatment condition, where no SSRI treatment was administered yet, limits insight into the effects of SSRIs.

Until now, the network approach has not been applied to explore the relationships among depressive symptoms before and after SSRI treatment. Such an approach may offer insight into the interrelations among symptoms before and after SSRI treatment and thus yield interesting directions for further study of how SSRIs may exert their effects. Therefore, the present study aimed to explore the possibilities of using cross-sectional network analysis before and after SSRI treatment by looking at the most recent and insightful network metrics. Specifically, we will examine (1) the interrelations, community structure, and connectivity of depression symptoms and (2) their centrality and predictability estimates in acutely depressed patients treated with the SSRI paroxetine, a commonly prescribed antidepressant. We combined the data of two large randomized controlled trials (RCTs) comparing paroxetine to pill-placebo and psychological treatment to retain sufficient power for the analyses. Focusing on the patients treated with paroxetine (*N* = 178), we constructed networks before the start of treatment and after 8 weeks of treatment. We compared the two resulting networks and their metrics to explore the possibilities of network analysis for increasing our understanding of symptom connectivity before and after SSRI treatment.

## Methods

### Participants

Patients with moderate to severe major depressive disorder participated in one of two similar RCTs for treatment of MDD in the United States, which were combined to increase statistical power [19, 20]. To be included, patients had to (1) fulfill the DSM-IV criteria for MDD, as assessed by the Structured Clinical Interview for the DSM-IV [21], (2) 18–70 years of age, (3) English speaking, (4) and willing and able to give informed consent. The first RCT, conducted at the University of Pennsylvania and Vanderbilt University (*N* = 240) between 1996 and 2001, only enrolled moderate to severely depressed patients (17-item Hamilton Rating Scale for Depression (HRSD-17) [22] ≥ 20). The second RCT, conducted at the University of Washington (*N* = 241), enrolled patients between 1998 and 2001 and included mildly, moderately, and severely depressed patients (Beck Depression Inventory (BDI-II) [23] ≥ 20 and HRSD-17 ≥ 14).

Patients were excluded in both trials if they had a lifetime diagnosis of psychosis, bipolar disorder, organic brain syndrome, or mental retardation. Further excluded were patients presenting with imminent suicide risk requiring immediate hospitalization, a current Axis I disorder judged to be primary, substance abuse, or antisocial, borderline, or schizotypal personality disorder. Lastly, participants who had not responded favorably to an adequate trial of either CT or paroxetine were excluded. The studies were conducted in accordance with the Declaration of Helsinki and institutional review boards of each of the three study sites approved the study protocols. All patients provided written informed consent.

### Treatment

**Fig. 1:**
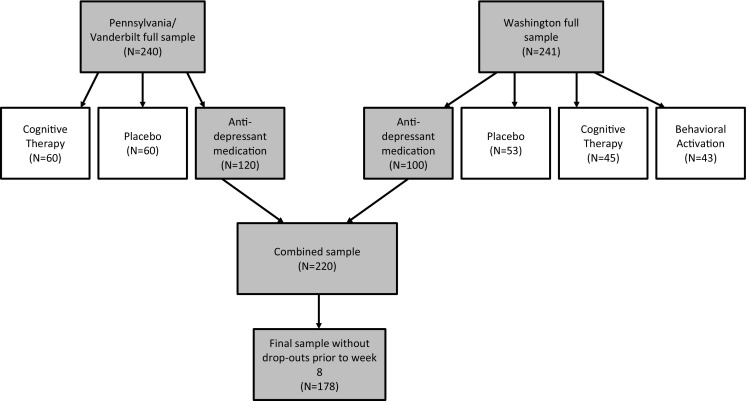
Overview of flow of participants throughout the two trials

The present study focuses on patients randomized to antidepressant medication (see Fig. 1), given our assumption that different treatments have different working mechanisms. Unfortunately, power was insufficient to construct separate networks for the other trial conditions. Patients randomized to one of the pill conditions were treated with the SSRI paroxetine or pill-placebo for 8 weeks in a double-blind manner. All patients receiving SSRI or pill-plabceo received weekly sessions with their prescribing clinician for the first 4 weeks and every other week thereafter. These sessions consisted of medication management, which involved education, adjustment of dosage and dosage schedules, and discussions of adverse effects, and of clinical management, which involved a review of the patient’s functioning in major life domains, brief supportive counseling, and limited advice giving [24]. The usage of specific psychotherapy techniques was not allowed.

### Measures

The BDI-II, a widely used self-report questionnaire with adequate psychometric properties [23, 25], was administered at baseline (before the start of treatment) and week 8 (mid-treatment). Each item is rated on a 4-point Likert scale, ranging from 0 to 3. Networks were constructed of 20 of the 21 individual items of the BDI-II at each time point. The BDI-II item on suicidal thoughts or wishes was removed from the network estimations given its low variance and positively skewed distribution [26] (see Supplementary Materials).

### Statistical analyses

#### Missing data

The individual SSRI treatment arms of each trial were not sufficiently large to estimate networks due to the large number of individual symptoms. We, therefore, combined the data from the Pennsylvania/Vanderbilt and Washington studies, resulting in a total of 220 patients randomized to SSRI treatment. Of those, 42 participants dropped out prior to week 8. To ensure both networks consisted of the same people, those 42 participants were removed from the network analyses, resulting in a final sample of 178 participants at both time points. Seven individuals had partially missing data. The Gaussian graphical models (GGM) were estimated on the full data set (*N* = 178) using pairwise complete observations (i.e., using all available information from all participants) [27]. Analyses of predictability and the network comparison test cannot deal with missing data, reducing the analytic data from 178 to 171 participants for those analyses.

#### Network estimation

First, we used the *R*-package *qgraph* (version 1.4.4) to estimate the network structures of the 20 BDI-II symptoms at baseline and at week 8 [28]. Networks contain *nodes* (symptoms) and *edges* (cross-sectional associations among symptoms). We estimated GGMs in which the edges represent partial correlation coefficients. To reduce false-positive edges, we applied the least absolute shrinkage and selection operator (lasso) [29]. This procedure penalizes very small edges by setting them to zero. The shrinkage parameter is chosen to minimize the extended Bayesian Information Criterion (EBIC) parameter [30] and has been shown to accurately recover underlying network structures [11]. We applied a conservative graphical tuning parameter of 0.5; false-positive edges are very unlikely, while very small actual edges may not be captured. Position of the nodes in the networks was initially based on the Fruchterman–Reingold algorithm, which places the nodes with stronger and/or more connections closer together [31]. We averaged the layout of the networks to facilitate visual comparison of the time points. To examine robustness of our findings, we compared the standard deviations (SDs) of the BDI-II sum score and all individual BDI-II items between the two time points by means of Levene’s test (corrected for chance capitalization by the conservative Bonferroni–Holm method). If SDs change significantly, differences in the network structure might be a result of increased variation [32].

#### Communities

Within the GGMs as specified above, we explored the way nodes within the networks cluster together through exploratory graph analysis (EGA) [33]. Nodes that cluster together in communities may be part of the same latent variable or dimension. EGA estimates communities in networks via a random walk algorithm (walktrap).

#### Connectivity

Network connectivity (or density) for each network was calculated by summing all absolute edge weights. The difference in network connectivity for baseline and week 8 was tested for significance via the R-package network comparison test (NCT), which uses permutation testing to compare networks [34].

#### Centrality

Strength centrality was calculated for all symptoms at baseline and week 8 by summing the absolute values of the edges of a given node to other nodes [10]. The higher the strength centrality, the more strongly is this symptom connected to other symptoms. The stability of strength centrality was calculated at baseline and week 8 through the correlation stability coefficient (CS-coefficient), which is a method based on bootstraps (N = 1000) using the R-package *bootnet* [35]. The CS-coefficient represents the maximum proportion of cases that can be dropped, such that with 95% probability the correlation between the original strength centrality estimates and the bootstrapped estimates is 0.7 or higher [35]. The higher the CS-coefficient, the more reliable the interpretation of the order of centrality estimates. The R-package *bootnet* was further used to test strength centrality among nodes for significance at baseline and week 8.

#### Predictability

We further estimated the predictability of each of the nodes in the network, which is an estimate of how much of the variance of a node is explained by neighboring nodes [36]. The R-package *mgm* [37] was used to estimate the proportion of explained variance for each node, which could range from zero (a node cannot be predicted by other nodes in the network) to one (a node is perfectly predicted by other nodes).

## Results

### Participant characteristics

Table 1 depicts the demographic and clinical characteristics of the two study samples and the combined sample. The RCT samples were found to be quite similar with a few exceptions indicating that the Pennsylvania/Vanderbilt study included a more severely depressed sample as is appropriate given the inclusion criteria. BDI-II scores at baseline did not differ significantly between the two studies and percent change of BDI-II sum-scores from baseline to week 8 was similar (Pennsylvania/Vanderbilt study: 56.4%; Washington study: 56.1%), as was the percent of missing data (Pennsylvania/Vanderbilt: 20.8%; Washington: 25%).

**Table 1:** Demographic and clinical characteristics before randomization to SSRI treatment

Variable	Combined sample (*N* = 170)	Pennsylvania/Vanderbilt study (*N* = 95)	University of Washington study (*N* = 75)	*P* value
Female, *N* (%)	93 (55%)	56 (59%)	54 (72%)	< 0.001
Caucasian, *N* (%)	140 (82%)	81 (85%)	59 (79%)	n.s
Age	40.0 (10.9)	40.7 (11.3)	39.1 (10.4)	n.s
Married/cohabitating, N (%)	66 (39%)	41 (43%)	25 (33%)	n.s
Employed, *N* (%)	135 (79%)	84 (88%)	51 (68%)	0.018
Chronic MDD, *N* (%)	85 (50%)	56 (59%)	29 (39%)	n.s
Age of onset	23.8 (12.5)	21.0 (12.4)	27.3 (11.9)	0.001
Number of previous episodes	1.9 (2.5)	2.7 (3.0)	1.0 (1.3)	< 0.001
Duration of current episode (months)	48.4 (74.5)	52.6 (77.1)	43.2 (71.2)	n.s
History of psychiatric hospitalization, *N* (%)	15 (9%)	10 (11%)	5 (7%)	n.s
Axis I comorbidity, *N* (%)	91 (54%)	71 (75%)	20 (27%)	< 0.001
Axis II comorbidity, *N* (%)	65 (38%)	50 (53%)	15 (20%)	< 0.001
BDI-II score baseline	31.6 (8.9)	31.2 (9.8)	32.1 (7.6)	n.s
BDI-II score week 8	13.8 (10.2)	13.6 (10.6)	14.1 (9.8)	n.s
HRSD-17 score baseline	22.3 (3.9)	23.8 (3.2)	20.4 (4.0)	< 0.001
HRSD-17 score week 8	12.3 (6.7)	12.4 (6.7)	12.2 (6.7)	n.s
Daily dosage of paroxetine baseline	12.2 (2.7)	14.0 (4.9)	10.0 (0)	–
Daily dosage of paroxetine week 8	35.7 (11.2)	38.8 (11.0)	31.7 (11.5)	–

Descriptive statistics represent mean (SD) unless otherwise stated. The table only includes individuals without missing data

*P* values represent tests between the two studies and were corrected for chance capitalization with the Bonferroni–Holm method

*BDI-II* Beck Depression Inventory-II, *HDRS-17* 17-item Hamilton Depression Rating Scale, *ns* non-significant

### Means and variation of all 21 BDI-II symptoms

In the combined sample, mean BDI-II sum score decreased significantly after 8 weeks by 44% (*W* = 26,052, *p* < .001). Further, as depicted in Table 2, mean scores for all individual BDI-II items decreased significantly as well (*p* values were corrected for chance capitalization via the conservative Bonferroni–Holm method). Loss of interest in sex (30% change) and appetite (38%) changed the least, whereas the largest changes were evident in suicidal ideation (74%), crying (72%), and feelings of punishment (70%). SD of the BDI-II sum score did not change significantly after 8 weeks of SSRI treatment (Levene’s test = 1.34, *p* = .25). Five SDs of the individual BDI-II items decreased significantly (punishment feelings, suicidality, crying, sleep, and irritability) and two increased (self-dislike and concentration difficulty).

**Table 2:** Overview of means and standard deviations of the individual BDI-II items for baseline and week 8 (*N* = 178)

Item	Abbreviation	Baseline		Week 8	
		Mean	SD	Mean	SD
Sadness	SAD	1.4	0.8	0.5	0.6
Pessimism	PES	1.4	0.8	0.7	0.7
Past failure	FAI	1.8	0.7	0.8	0.8
Loss of pleasure	LPLE	1.8	0.7	0.9	0.8
Guilty feelings	GUI	1.3	0.8	0.5	0.6
Punishment feelings	PUN	1.0	1.1	0.3	0.7
Self-dislike	DIS	1.9	0.8	0.9	0.9
Self-criticalness	CRI	1.7	0.9	0.7	0.7
Suicidal thoughts or wishes*		0.7	0.6	0.2	0.4
Crying	CRY	1.6	1.1	0.5	0.9
Agitation	AGI	1.0	0.9	0.5	0.7
Loss of interest	LINT	1.9	0.9	0.8	0.8
Indecisiveness	IND	1.8	1.0	0.7	0.8
Worthlessness	WOR	1.6	0.8	0.5	0.7
Loss of Energy	LENE	1.7	0.8	0.9	0.8
Change in sleep	SLE	1.4	1.1	0.8	0.9
Irritability	IRR	1.5	0.9	0.5	0.7
Change in appetite	EAT	0.9	0.9	0.6	0.7
Concentration difficulty	CON	1.8	0.6	0.8	0.8
Fatigue	FAT	1.9	0.9	0.9	0.8
Loss of interest in sex	LSEX	1.5	1.0	1.0	1.1

*Because of its problematic distribution and small standard deviation, this BDI-II item was not included in the network analyses

### Network connectivity and community detection

**Fig. 2:**
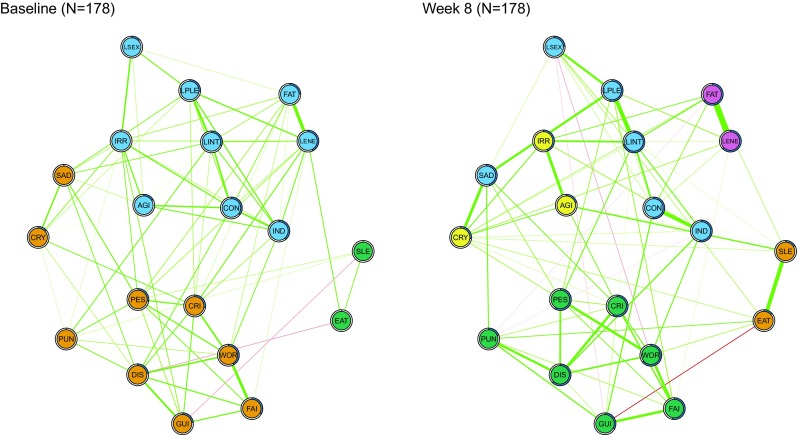
EBIC gLasso network of BDI-II symptoms before the start of treatment (left) and after 8 weeks of paroxetine treatment (N = 178). Note: Abbreviations can be found in Table 2. Green lines represent a positive association between two symptoms. The thicker the edge (line), the stronger the relationship between two symptoms. The color of the nodes represents the community the symptom belongs to; nodes with similar color belong to the same community. The proportion of explained variance (predictability) can be derived from the blue ring surrounding the node

Figure 2 displays the GGM networks of the 20 BDI-II symptoms at baseline and week 8 (*N* = 178). The network for week 8 showed significantly higher connectivity: the sum of all edges was 9.1 compared to 7.3 for the baseline network (*p* = 0.02).

At baseline, three symptom communities emerged: (1) a cluster of cognitive symptoms such as worthlessness, but also sad mood (orange nodes), (2) an affective behavioral cluster of symptoms such as loss of pleasure, energy, and agitation (blue), and (3) a cluster of sleep and appetite symptoms (green).

After 8 weeks of SSRI treatment, five communities emerged. The sleep and appetite cluster remained (orange nodes). The cognitive cluster also remained but lost symptoms of sad mood and crying (green). Three other clusters were identified: a cluster of irritation, agitation, and crying (yellow), a cluster of fatigue and loss of energy (pink), and an affective behavioral cluster of symptoms such as sad mood, loss of interest, and agitation (blue).

### Symptom centrality and predictability

**Fig. 3:**
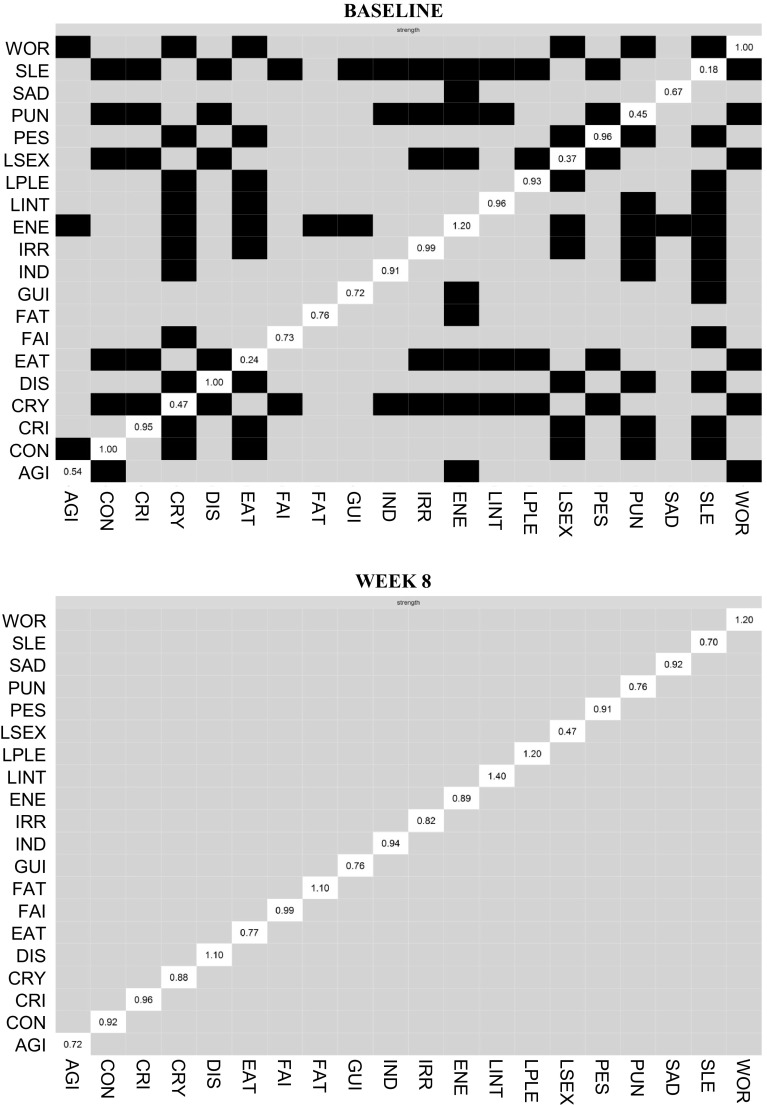
Bootstrapped difference tests of strength centrality between nodes within the baseline network and the week 8 network (*N* = 178). Note: Gray boxes indicate nodes that did not significantly from one another and black boxes indicate nodes that do differ significantly from one another. White boxes show the value of strength centrality for a given node

Figure 3 shows bootstrapped difference tests of strength centrality between nodes within the baseline network and the week 8 network (*N* = 178). At baseline, the most central symptom was loss of energy. Loss of energy showed a significantly higher strength centrality than nine other symptoms. In contrast, sleep had the lowest strength centrality and was significantly more weakly connected than 12 other symptoms. Mean severity of the symptoms was strongly associated with strength centrality (*r* = 0.69, *p* < 0.001), indicating that symptoms with higher severity were also more strongly interconnected.

At week 8, sleep remained the most weakly connected symptom; the most central symptom was loss of interest. However, no symptoms showed significantly stronger connections than other nodes in the network. Strength centrality (*r* = 0.33, *p* = 0.23) was no longer associated with mean symptom severity. The CS-coefficient was 0.13 for both baseline and week 8. This indicates the centrality estimates need to be interpreted with caution.

Finally, the overall proportion of explained variance (predictability) of nodes increased significantly from 0.21 at baseline to 0.45 at week 8 (*t* = − 8.6, *p* < 0.001). At baseline, worthlessness and loss of energy showed the largest predictability relative to the other nodes; whereas for week 8, these were loss of interest and fatigue. The correlation between the predictability of the two time points was 0.76 (*p* < 0.001), indicating that if a node has high predictability at baseline, it tends to also have a high predictability at week 8. As expected, strength centrality was strongly related to the predictability of nodes at baseline (*r* = 0.73, *p* < 0.001) and week 8 (*r* = 0.80, *p* < 0.001), suggesting that if a node is strongly connected to other variables, it tends to be predicted by other nodes as well. Finally, although predictability was related to mean symptom severity at baseline (*r* = 0.68, *p* < 0.001), they were not significantly related at week 8 (*r* = 0.44, *p* = 0.053).

## Discussion

This study examined changes in the relationships among depressive symptoms after 8 weeks of SSRI treatment in a sample of outpatients treated for moderate to severe depression. To our knowledge, this is the first study to compare network structures before and after SSRI treatment.

The severity of all individual depressive symptoms significantly decreased after 8 weeks of paroxetine treatment and they became more strongly associated. The latter finding contrasts with previous network studies reporting that increased network connectivity is associated with acute depression [14, 15], but is consistent with findings of increased network connectivity paralleling decreases in symptom severity [16, 38, 39]. Corresponding to a large body of literature on the factor solution of the BDI-II [13, 23, 40, 41], we found a cluster consisting of cognitive symptoms both at baseline and week 8. However, the affective behavioral cluster usually also reported was split into several separate clusters in our sample, which also differed somewhat across the two time points.

Based on network theory, one would expect network connectivity to decrease as symptoms are alleviated; when symptoms become more weakly connected, they may be less easily triggered by other symptoms [7]. Our findings that network connectivity increased after depressive symptoms decreased during SSRI treatment, therefore, seems counterintuitive. However, previous studies with contrasting findings used between-subject rather than within-subject designs; van Borkulo et al. compared baseline networks of participants with persistent depression to participants who later remitted [14], and Pe et al. compared depressed patients versus healthy controls using dynamic data [15]. The present study examined connectivity over time within the same participants, which may explain the difference in results.

Indeed, our pattern of results, increased network connectivity as symptom severity decreased, is highly consistent with previous studies reporting a different network structure paralleling a decrease in symptom severity [16, 38, 39]. Fried et al. have suggested this pattern of results may have several possible explanations [16]. First, interpretation of items (of the BDI-II) may change due to psychological treatment, which is termed response bias. This seems less likely in our sample, given that our sample did not receive psychological treatment. Second, variability of symptoms may change over time, explaining differences in network connectivity [32]. Although the standard deviations of the BDI-II individual items did not change significantly for 14 of the 20 items used in the network analyses, the proportion of increased variance (predictability) did increase at week 8. Finally, the hypothesis that increased network connectivity may be the result of SSRI treatment seems less likely, given parallel findings in other samples in which not all participants were on antidepressant medication [16, 38, 39]. Unfortunately, the lack of a control group in the present study precludes insight into potential unique effects of SSRIs on network connectivity.

Another aim of this study was to examine changes in the order of strength centrality after 8 weeks of SSRI treatment. This would enable us to investigate if and how SSRIs change the way symptoms are related to one another. However, due to the small sample size, power was too low to detect significant differences in the ordering of strength centrality at the two time points, especially at week 8. Indeed, correlation stability coefficients (0.13) were lower than the recommended lower bound of 0.25 [35], suggesting that our estimation of the ordering of the centrality of symptoms may be less reliable. Therefore, interpretation of the order of symptoms remains speculative.

However, at baseline, some symptoms appear to be significantly more central than others, indicating they are more strongly connected. Indeed, our finding that loss of energy had the highest strength centrality at baseline, significantly higher than nine other symptoms, is not completely surprising. The high centrality of loss of energy in depressed patients has been found in other network studies [11, 13, 42] and is in line with evolutionary theory, which proposes that depressive symptoms force an individual to conserve energy during adverse situations and reallocate it to solving the problem [43, 44]. In a prospective study, strength centrality of fatigue was a strong predictor of depression onset [42]. It is speculated that by targeting and reducing central symptoms, because of its strong connections, other symptoms are reduced in parallel, which may prove to be a valuable treatment strategy [45]. However, other explanations may be possible as well, such as that highly central symptoms are part of the same latent variable (for example, loss of energy and fatigue may tap into the same construct), and because they are so strongly interconnected, all estimates of strength centrality are biased [46].

The centrality of loss of energy decreased relative to the other symptoms at week 8; instead, loss of interest became the most central symptom. However, since strength centrality was not significantly higher for loss of interest than any other symptom, we cannot conclude with certainty that it is indeed more central. If our findings are replicated, a speculative thought may be that SSRIs weaken the connections of energy loss to other depressive symptoms, thereby alleviating depression. The finding that loss of interest became the most central symptom may suggest that if anhedonia is still present after treatment, other symptoms are still present too. Indeed, studies have shown that symptoms of anhedonia are resistant to SSRI treatment and predictive of poorer outcome [47–49]. SSRIs do not appear to target dopamine systems; thus they might not target symptoms related to the experience of positive affect and reward [50, 51]. Speculatively, SSRIs may sever the links between symptoms related to negative affect and energy, whereas the relationships between anhedonia and other symptoms remain. However, power issues and lack of a comparison group prevented us from elucidating specific effects of SSRI treatment on the strength centrality of symptoms.

Important strengths of our study include the use of state-of-the-art network analysis techniques, our specific investigation of one type of SSRI, and the study design, which enabled us to compare symptom networks before and after treatment. However, our findings should also be considered in light of several limitations. First, because of insufficient power, we were not able to take advantage of the placebo controls. Thus, any observed change may not be due to specific effects of the SSRI. Second, our sample size was suboptimal for a network analysis with such a large number of nodes, limiting insight into potential significant differences in symptom centrality. Third, the positive skewness of the individual BDI-II items at week 8 may have affected our results [32]. Future investigations into how skewness should be addressed in Gaussian network models are warranted. Fourth, the specific characteristics of our sample may have limited generalizability. Also, BDI-II sum score was used as an inclusion criterion. Using the same instrument for inclusion and network analyses may have influenced the results [52], although we expect this effect to be small given that participants were also selected based on other instruments (HRSD, SCID-I). Given these limitations, future studies with larger sample sizes and placebo controls are necessary before conclusions on the effects of SSRI on symptom interrelations can be drawn.

A final note on the interpretation of cross-sectional networks should be made considering recent concerns on the replicability of networks [53]. Indeed, the network literature will benefit from insight into whether reported network structures replicate across data sets before clinical inferences can be made. First attempts to study replicability have thus far yielded conflicting results (see [53–55]). Furthermore, cross-sectional networks face the limitation that they may only reveal the co-occurrence of symptoms, not how they follow each other over time [56], and it has been questioned whether cross-sectional group-level associations can be generalized to the level of the individual [57]. Our findings, therefore, need to be replicated, preferably by designs that offer more insight into the temporal dynamics of relationships between symptoms at the individual level [58]. Specifically, such temporal data enable investigation into changes in which symptoms precede changes in other symptoms. Consequently, one can be more certain about the direction of change, allowing the identification of symptoms that precede change in other symptoms [13]. We, therefore, consider this study an exploration of the possibilities of applying the network approach to uncover novel insights into the way antidepressant treatment works. Follow-up studies are required to examine whether central symptoms and changes over time generalize to the dynamics of symptoms taking place within individual patients.

To our knowledge, this was the first study to examine changes in symptom interrelations and their centrality after 8 weeks of SSRI treatment. Our findings demonstrated an increase in the interrelations between depressive symptoms after SSRI treatment, which adds to previous research reporting increased connectivity after symptom severity decreased. The current findings highlight the potential of the network approach in providing insight into the working mechanisms of SSRIs on the interrelations between depressive symptoms, which should be explored in future research.

## Electronic supplementary material

Below is the link to the electronic supplementary material.

Supplementary material 1 (PDF 116 KB)
