# Supplementary material for: Cross-sectional networks of depressive symptoms before and after antidepressant medication treatment
Source: Soc Psychiatry Psychiatr Epidemiol. 2018 Apr 7;53(6):617–27. doi: 10.1007/s00127-018-1506-1 (PMC5959987; doi:10.1007/s00127-018-1506-1)
Supplement: Supplementary file 1 — Supplementary material 1 (PDF 116 KB) [file 127_2018_1506_MOESM1_ESM.pdf]

Supplementary materials for:

Cross-sectional networks of depressive symptoms before and after antidepressant medication treatment

RUNNING HEAD: NETWORKS BEFORE AND AFTER ANTIDEPRESSANTS

Fionneke M. Bos[1,2*], M.Sc.

Eiko I. Fried[3], PhD

Steven D. Hollon[4], PhD

Laura F. Bringmann[3], PhD

Sona Dimidjian[5], PhD

Robert J. DeRubeis[2], PhD

Claudi L.H. Bockting[7], PhD

[1] University of Groningen, University Medical Center Groningen, Department of Psychiatry, Rob Giel Research Center, Department of Psychiatry, Groningen, The Netherlands

[2] Department of Psychology, University of Pennsylvania, Philadelphia, Pennsylvania, United States of America

[3] Department Quantitative Psychology and Individual Differences, University of Leuven, Leuven, Belgium

[4] Department of Psychology, Vanderbilt University, Nashville, Tennessee, United States of America

[5] Department of Psychology and Neuroscience, University of Colorado Boulder, Boulder, Colorado, United States of America

[7] Department of Clinical Psychology, Utrecht University, Utrecht, The Netherlands

* Corresponding author: Fionneke Bos, MSc, University of Groningen, University Medical Center Groningen, Department of Psychiatry, Rob Giel Research Center, PO Box 30.001, 9700 RB, Groningen, The Netherlands. Phone: +31503615725, e-mail: f.m.bos01@umcg.nl. Orcid: 0000-0002-9630-0440

1. Distribution of individual BDI-II items

The distribution of all individual items of the Beck Depression Inventory II can be found on the following pages.

Baseline − Sadness

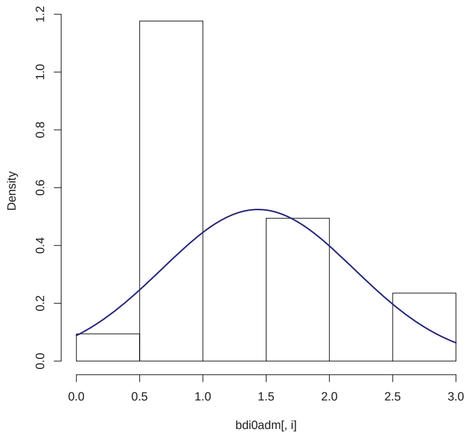

Baseline − Pessimism

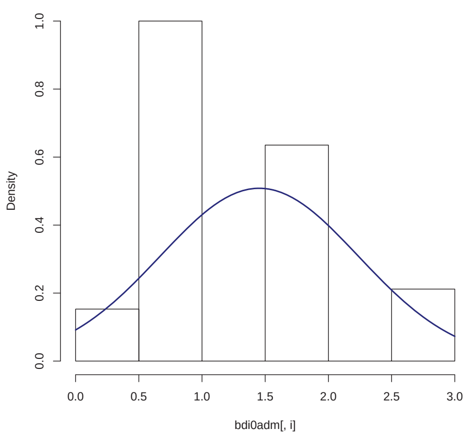

Baseline − Past Failure

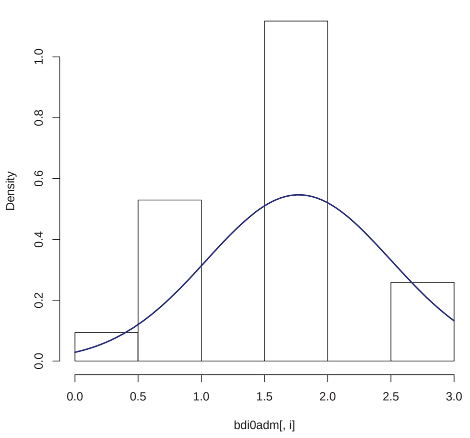

Baseline − Loss of Pleasure

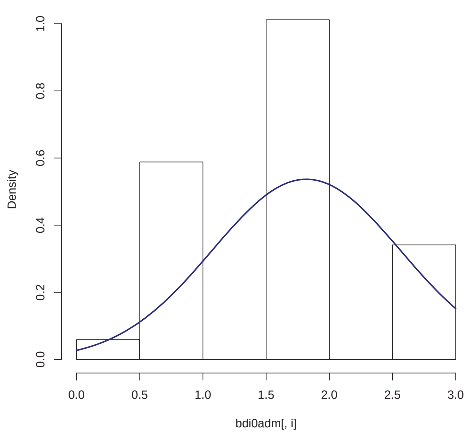

Baseline − Guilty Feelings

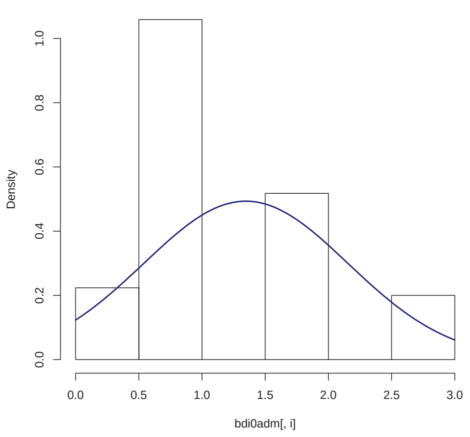

Baseline − Punishment Feelings

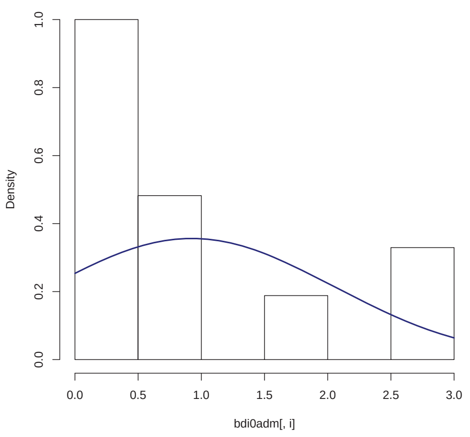

Baseline − Self Dislike

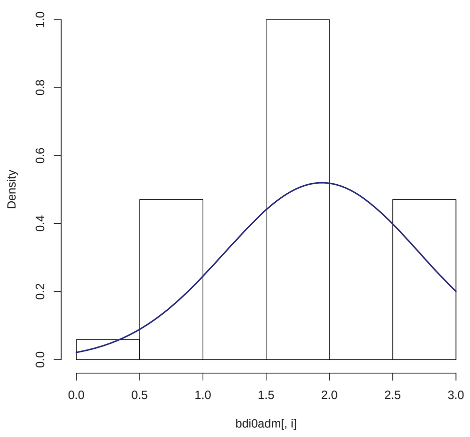

Baseline − Self Criticalness

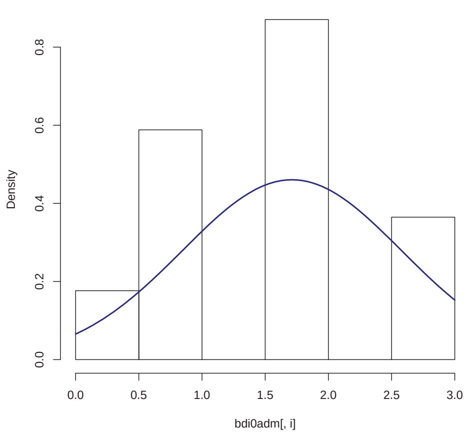

Baseline − Crying

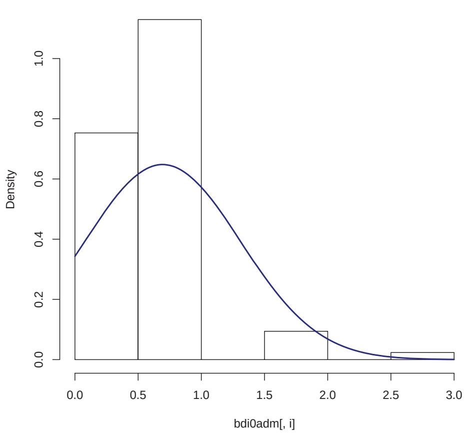

Baseline − Agitation

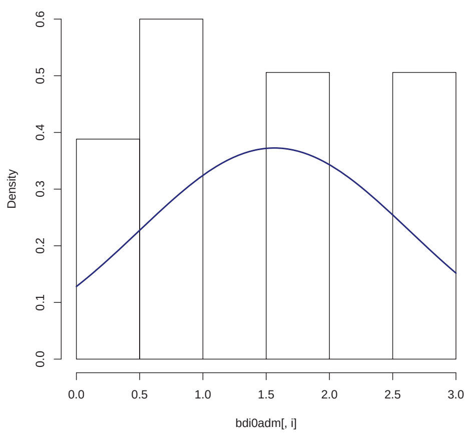

Baseline − Loss of Interest

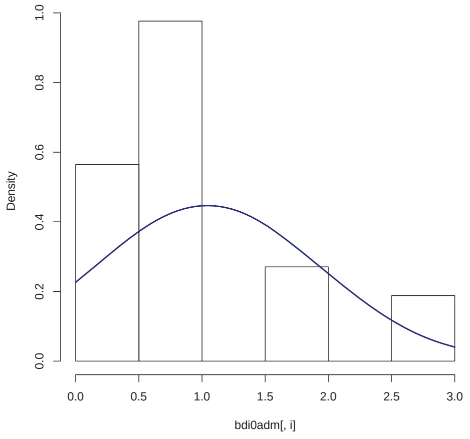

Baseline − Indecisiveness

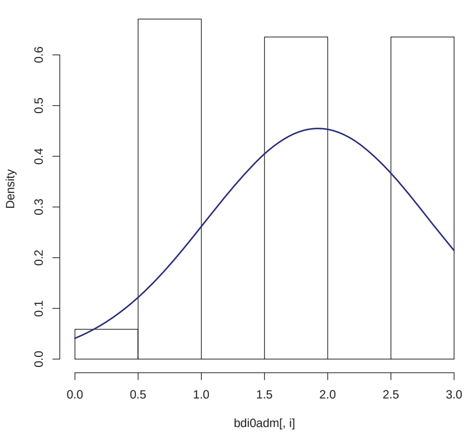

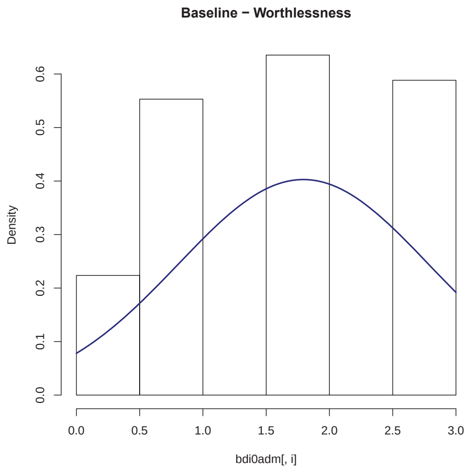
Baseline − Worthlessness
Density
0.0 0.1 0.2 0.3 0.4 0.5 0.6
0.0 0.5 1.0 1.5 2.0 2.5 3.0
bdi0adm[, i]

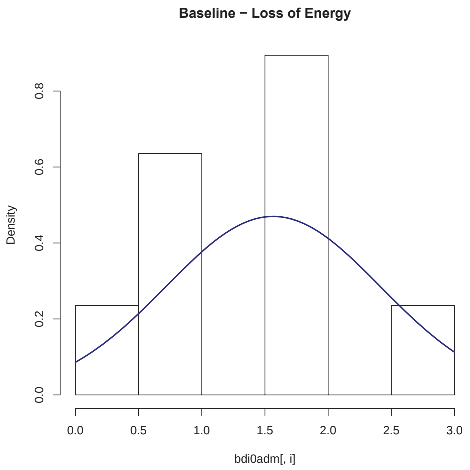
Baseline − Loss of Energy
Density
0.0 0.2 0.4 0.6 0.8
0.0 0.5 1.0 1.5 2.0 2.5 3.0
bdi0adm[, i]

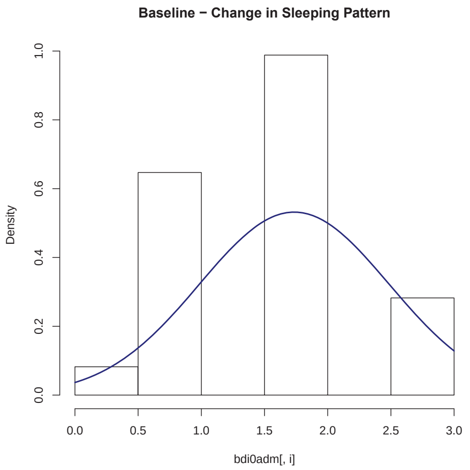
Baseline − Change in Sleeping Pattern
Density
0.0 0.2 0.4 0.6 0.8 1.0
0.0 0.5 1.0 1.5 2.0 2.5 3.0
bdi0adm[, i]

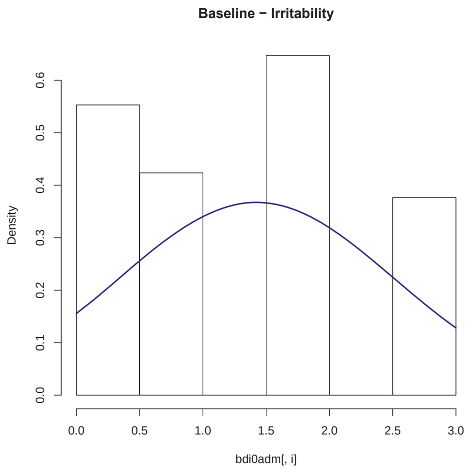
Baseline − Irritability
Density
0.0 0.1 0.2 0.3 0.4 0.5 0.6
0.0 0.5 1.0 1.5 2.0 2.5 3.0
bdi0adm[, i]

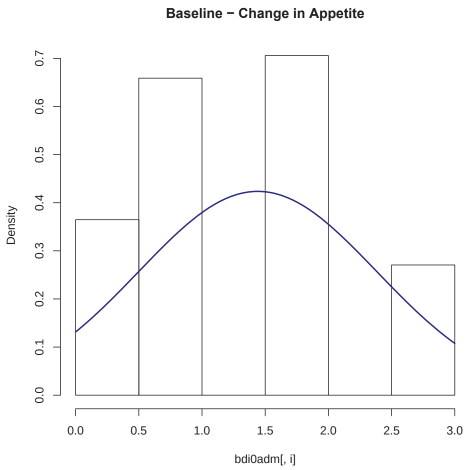
Baseline − Change in Appetite
Density
0.0 0.1 0.2 0.3 0.4 0.5 0.6 0.7
0.0 0.5 1.0 1.5 2.0 2.5 3.0
bdi0adm[, i]

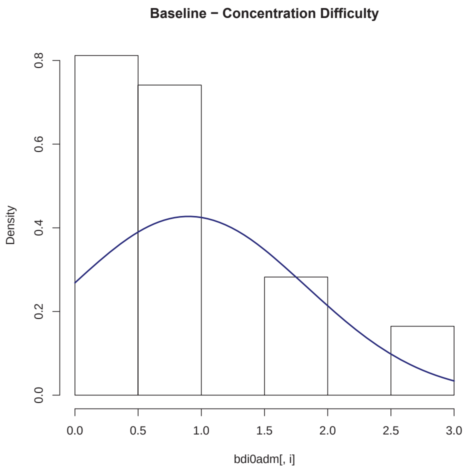
Baseline − Concentration Difficulty
Density
0.0 0.2 0.4 0.6 0.8
0.0 0.5 1.0 1.5 2.0 2.5 3.0
bdi0adm[, i]

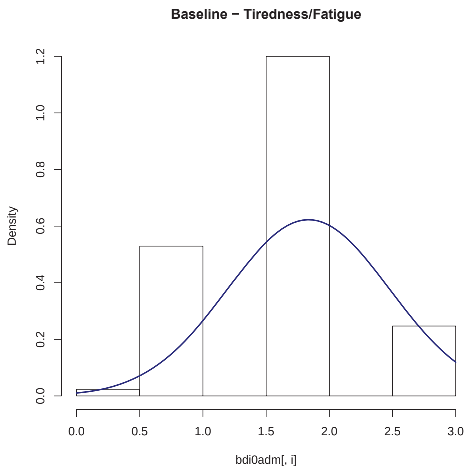
Baseline − Tiredness/Fatigue
Density
0.0 0.2 0.4 0.6 0.8 1.0 1.2
0.0 0.5 1.0 1.5 2.0 2.5 3.0
bdi0adm[, i]

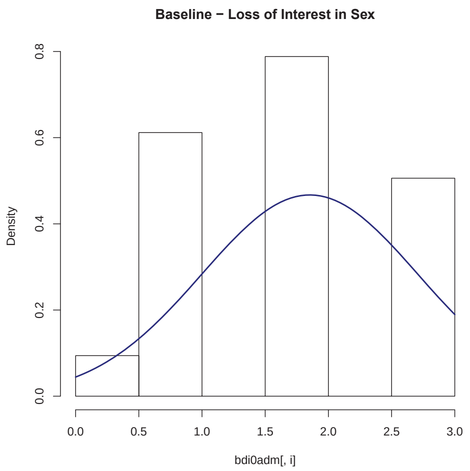
Baseline − Loss of Interest in Sex
Density
0.0 0.2 0.4 0.6 0.8
0.0 0.5 1.0 1.5 2.0 2.5 3.0
bdi0adm[, i]

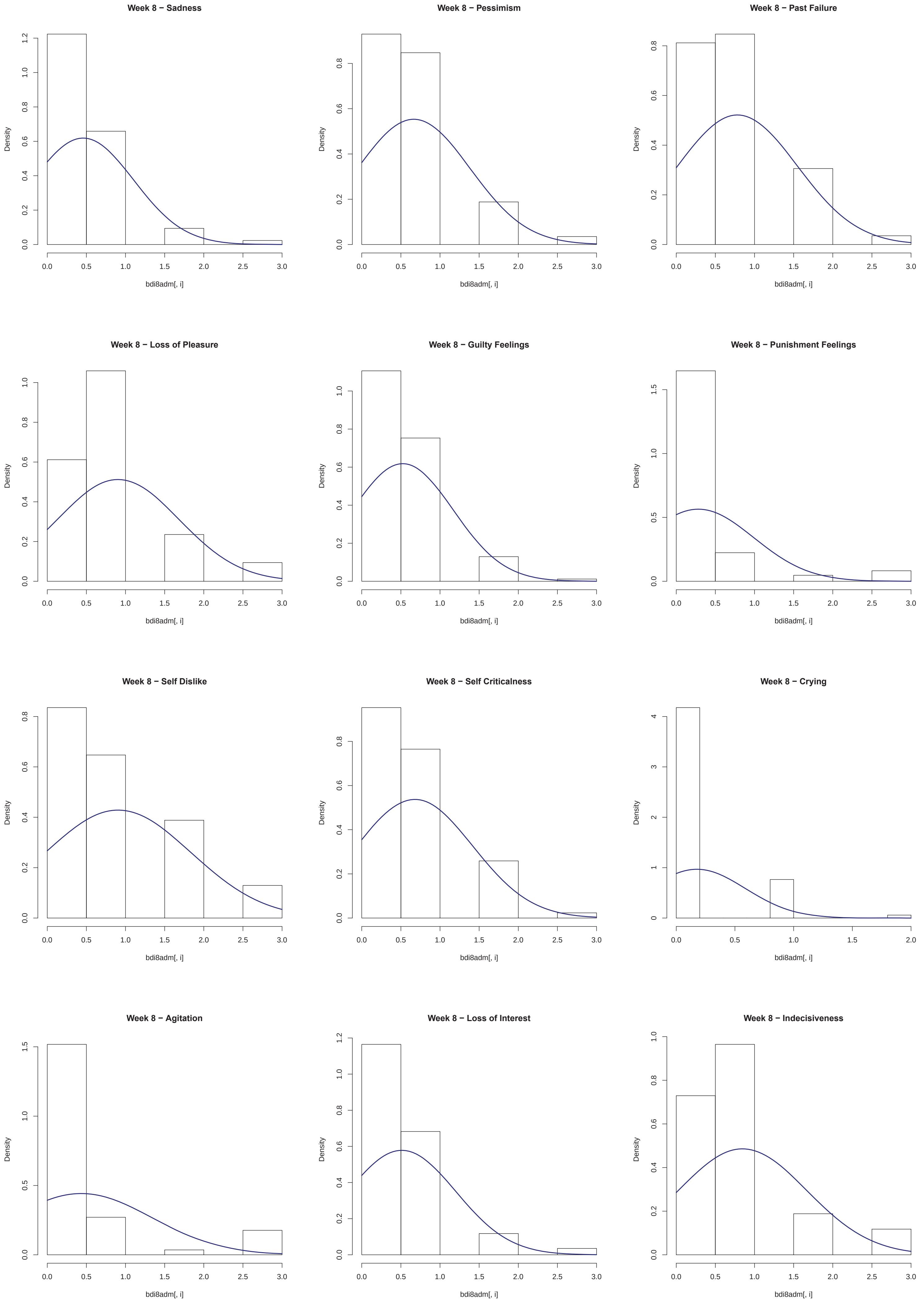
Week 8 − Sadness
Week 8 − Pessimism
Week 8 − Past Failure
Week 8 − Loss of Pleasure
Week 8 − Guilty Feelings
Week 8 − Punishment Feelings
Week 8 − Self Dislike
Week 8 − Self Criticalness
Week 8 − Crying
Week 8 − Agitation
Week 8 − Loss of Interest
Week 8 − Indecisiveness
Density
bdi8adm[, i]

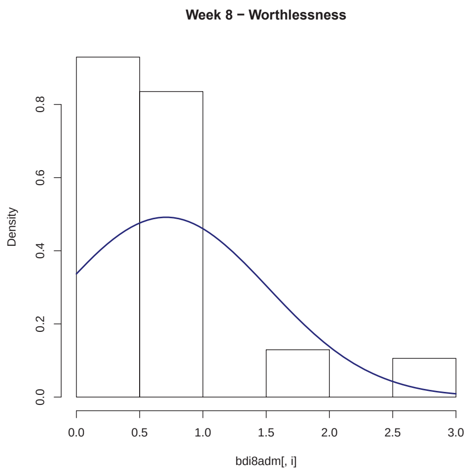
Week 8 − Worthlessness
Density
bdi8adm[, i]

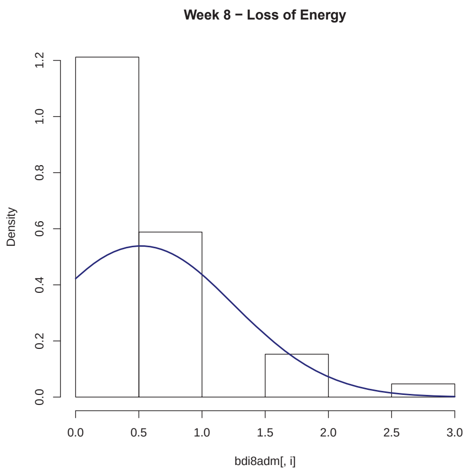
Week 8 − Loss of Energy
Density
bdi8adm[, i]

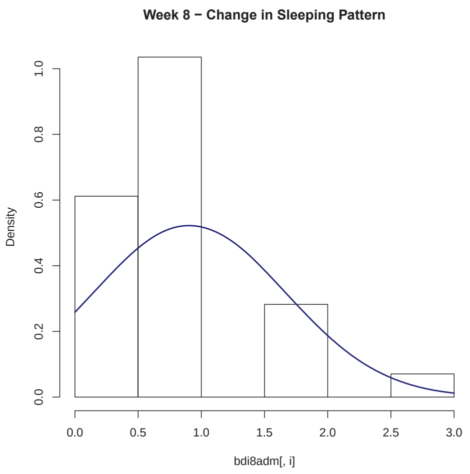
Week 8 − Change in Sleeping Pattern
Density
bdi8adm[, i]

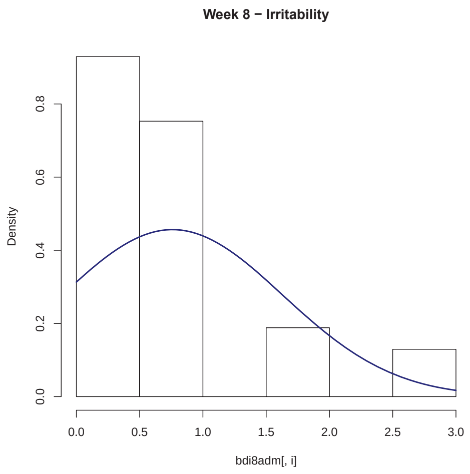
Week 8 − Irritability
Density
bdi8adm[, i]

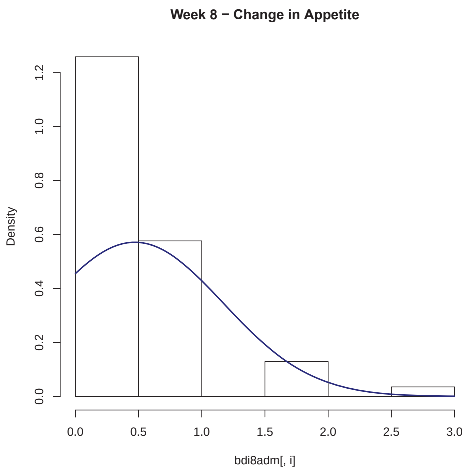
Week 8 − Change in Appetite
Density
bdi8adm[, i]

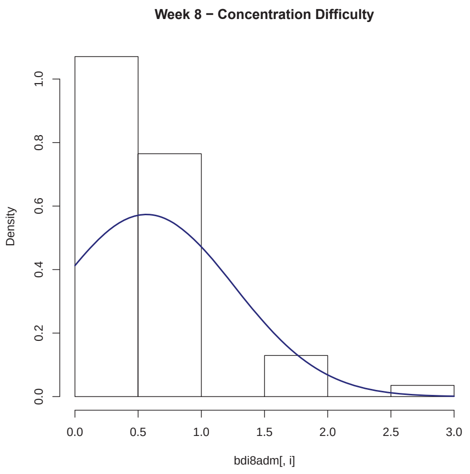
Week 8 − Concentration Difficulty
Density
bdi8adm[, i]

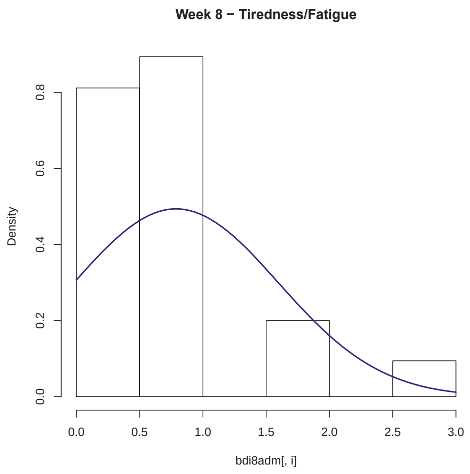
Week 8 − Tiredness/Fatigue
Density
bdi8adm[, i]

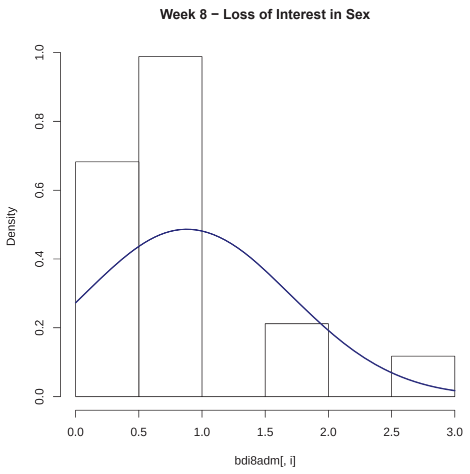
Week 8 − Loss of Interest in Sex
Density
bdi8adm[, i]
